# Emerging disease issues and fungal pathogens associated with HIV infection.

**DOI:** 10.3201/eid0202.960205

**Published:** 1996

**Authors:** N M Ampel

**Affiliations:** University of Arizona College of Medicine, Tuscon Veterans Affairs Medical Center, Tucson, Arizona 85713, USA. namp@aol.com

## Abstract

Fungal diseases are increasing among patients infected with human immunodeficiency virus (HIV) type 1. Infections due to Candida and Cryptococcus are the most common. Although mucocutaneous candidiasis can be treated with oral antifungal agents, increasing evidence suggests that prolonged use of these drugs results in both clinical and microbiologic resistance. The optimal therapy for cryptococcal meningitis remains unresolved, although initial treatment with amphotericin B, followed by life-long maintenance therapy with fluconazole, appears promising. Most cases of histoplasmosis, coccidioidomycosis, and blastomycosis occur in regions where their causative organisms are endemic, and increasing data suggest that a significant proportion of disease is due to recent infection. Aspergillosis is increasing dramatically as an opportunistic infection in HIV-infected patients, in part because of the increased incidence of neutropenia and corticosteroid use in these patients. Infection due to Penicillium marneffei is a rapidly growing problem among HIV-infected patients living in Southeast Asia. Although the advent of oral azole antifungal drugs has made primary prophylaxis against fungal diseases in HIV-infected patients feasible, many questions remain to be answered before the preventive use of antifungal drugs can be advocated.


### Emerging Disease Issues and Fungal Pathogens Associated with HIV Infection

**Neil M. Ampel, M.D.**
University of Arizona College of Medicine, Tucson Veterans Affairs Medical Center, Tucson, Arizona, USA

Fungal diseases are increasing among patients infected with human immunodeficiency virus (HIV) type 1. Infections due to *Candida* and *Cryptococcus* are the most common. Although mucocutaneous candidiasis can be treated with oral antifungal agents, increasing evidence suggests that prolonged use of these drugs results in both clinical and microbiologic resistance. The optimal therapy for cryptococcal meningitis remains unresolved, although initial treatment with amphotericin B, followed by life-long maintenance therapy with fluconazole, appears promising. Most cases of histoplasmosis, coccidioidomycosis, and blastomycosis occur in regions where their causative organisms are endemic, and increasing data suggest that a significant proportion of disease is due to recent infection. Aspergillosis is increasing dramatically as an opportunistic infection in HIV-infected patients, in part because of the increased incidence of neutropenia and corticosteroid use in these patients. Infection due to *Penicillium marneffei* is a rapidly growing problem among HIV-infected patients living in Southeast Asia. Although the advent of oral azole antifungal drugs has made primary prophylaxis against fungal diseases in HIV-infected patients feasible, many questions remain to be answered before the preventive use of antifungal drugs can be advocated.

Over the last decade, the incidence of fungal infections has increased dramatically. The human immunodeficiency virus (HIV) type 1 epidemic accounts for a large share of this increase. This article is not a general guide to the diagnosis or treatment of fungal diseases but rather a review of emerging disease issues in regard to these infections among HIV-infected patients. The infections discussed include candidiasis; cryptococcosis; the endemic mycoses histoplasmosis, coccidioidomycosis, and blastomycosis; aspergillosis; and penicilliosis. In addition, the role of preventive therapy for fungal infections in HIV-infected patients is assessed. Fungal pathogens and their most common manifestations in HIV-infected patients are listed separately (Table).

### Candidiasis

Mucocutaneous candidiasis is one of the most common manifestations of HIV infection. In one prospective study, 84% of HIV-infected patients had oropharyngeal colonization by *Candida* species on at least one occasion, and 55% developed clinical thrush (1). While other yeasts may occasionally cause clinical disease, *Candida albicans* is the organism isolated from most patients (1, 2). *Candida* species normally colonize the gastrointestinal tract of healthy adults, and most infections in HIV-infected patients are endogenously acquired. In some cases, candidal strains can be transmitted from person to person (1).

Table. Common fungal pathogens in HIV infection and their most frequent clinical syndromes

| Organism | Clinical syndrome |
|---|---|
| *Candida albicans* | Thrush, vaginal candidiasis, esophageal candidiasis |
| *Cryptococcus neoformans* | Meningitis |
| *Histoplasma capsulatum* | Disseminated infection with fever and weight loss |
| *Coccidioides immitis* | Diffuse and focal pulmonary disease |
| *Blastomyces dermatitidis* | Localized pulmonary disease and disseminated infection, including meningitis |
| *Aspergillus fumigatus* | Pulmonary disease with fever, cough, and hemoptysis |
| *Penicillium marneffei* | Fever alone or with pulmonary infiltrates, lymphadenopathy, or cutaneous lesions |

Address for correspondence: Neil M. Ampel, M.D., Medical Service (111), Veterans Affairs Medical Center, 3601 S. Sixth Avenue, Tucson, AZ 85713, USA; fax: 520-629-1861; e-mail: nampl@aol.com.

During the course of HIV infection, patients appear to be colonized with one or a few dominant strains, which tend not to change over time. Powderly and colleagues isolated the same strain of *C. albicans* in 11 of 17 patients with recurrent yeast infection, by DNA probe analysis (2). In another study, using contour-clamped homogeneous electric field electrophoresis, Sangeorzan et al. found that 60% of patients were colonized with one dominant strain of *C. albicans*. In 74% of these patients, recolonization with the same strain occurred after antifungal therapy (1). Using biotyping and restriction fragment length polymorphism analysis of 25S ribosomal DNA, Whelan and colleagues found that strains of *C. albicans* isolated from 24 patients with AIDS were not significantly different from strains from 23 patients without HIV infection (3). Thus, the candidal strains causing disease in patients with HIV infection appear to be the same as those colonizing patients without HIV infection and, in most patients, do not change over time.

In HIV-infected patients, candidiasis is virtually always mucocutaneous, involving the oropharynx, the esophagus, and the vagina. HIV infection by itself is not associated with the syndrome of disseminated candidiasis, which is characterized by candidemia, endophthalmitis, and multiple organ involvement. The precise immunologic processes that control candidal infection in HIV-infected patients are not known. However, mucocutaneous candidiasis is clearly related to the development of clinical cellular immunodeficiency. In fact, oropharyngeal candidiasis is an independent predictor of immunodeficiency in patients with AIDS (4). Moreover, a CD4 lymphocyte count < 200/µl is a major risk factor for the development of clinical thrush in HIV-infected persons (1).

Although oropharyngeal candidiasis is frequent in men, recurrent vaginal candidiasis is a common early manifestation of HIV infection in women. The location and severity of candidiasis in women with HIV infection appear to be closely associated with the degree of cellular immunodeficiency, based on the peripheral blood CD4 lymphocyte count. In a study of 66 women, mucocutaneous candidiasis developed in more than half of the women over a median of 14 months of follow-up; vaginal candidiasis, with a mean CD4 lymphocyte count of 506/µl, developed only in 10, while oropharyngeal candidiasis, with a mean count of 230/µl developed in 16, and esophagitis, with a mean count of 30/µl developed in nine (5).

Mucocutaneous candidiasis can be treated either topically or with systemic antifungal agents (1, 6), but such therapy does not eradicate colonization (1). Recently, several reports have noted the failure of azole drugs, particularly fluconazole, to treat recurrent cases of oropharyngeal candidiasis (1, 7). While factors such as diminishing cellular immunity, drug interactions, or decreased drug absorption may account for some of these treatment failures, increasing evidence suggests that *Candida* organisms are developing drug resistance.

In the past, a lack of consensus on the methods for performing antifungal susceptibility tests made it difficult to establish whether clinical failure of antifungal therapy was due to resistance of the organism. However, the National Committee for Clinical Laboratory Standards (NCCLS) has now developed reference methods allowing for uniform testing of yeast isolates (8). Using an NCCLS method, Sangeorzan et al. found that the MIC of fluconazole for *Candida* isolates increased over time among patients who had received fluconazole compared with those who received clotrimazole. Clinical resistance to fluconazole was associated with an increased MIC required by the isolate as well as the patient's low CD4 lymphocyte count (1). These data strongly suggest that continued use of antifungal agents, particularly fluconazole, leads to both clinical treatment failure and antifungal resistance, especially in highly immunodeficient patients.

### Cryptococcosis

A rare disease before the HIV epidemic, cryptococcosis was identified very early in the epidemic as one of the most common life-threatening infections in AIDS patients (9). However, issues regarding its epidemiology and therapy remain unresolved.

A single species, *Cryptococcus neoformans*, is responsible for virtually all clinical cases of cryptococcosis. The species exists in two varieties, *neoformans* and *gattii*, which inhabit different ecologic niches. *C. neoformans* var. *neoformans* has been isolated in many parts of the world from numerous sites, most frequently from soil containing high amounts of dried bird excreta, particularly of pigeons and chickens. It has long been presumed that inhalation of soil contaminated with such excreta is the most likely source of cryptococcal infection. However, few data support this hypothesis. In contrast, the only known environmental source of *C. neoformans* var. *gattii* is the river red gum tree (*Eucalyptus camaldulensis*), which grows in rural Australia. Although infection due to var. *neoformans* is worldwide, cases due to var. *gattii* have only been identified in tropical and subtropical regions, including areas where *E. camaldulensis* is not found (10).

Virtually all instances of cryptococcosis among HIV-infected persons have been caused by var. *neoformans*. The ubiquity of var. *neoformans* in the environment may be making exposure and subsequent infection likely; however, no clear link has ever been established between environmental sources of *C. neoformans* and the development of cryptococcosis in patients with HIV infection (10). In a recent study, Varma and colleagues, using genomic probe analysis, could not find a direct link between environmental sources of *C. neoformans* and infection in patients with AIDS or a unique strain of *C. neoformans* that was infecting these patients (11). Suppression of cellular immunity appears to be a critical factor in the development of cryptococcosis in HIV-infected patients, with the development of disease relating directly to the risk for AIDS and to the CD4 lymphocyte count (12, 13).

The appropriate therapy for cryptococcal meningitis in HIV-infected patients is unsettled at this time. The combination of amphotericin B plus flucytosine for 4 to 6 weeks has been considered standard for patients without HIV infection. However, concern about increased toxicity and decreased efficacy (12) has led to a reconsideration of this regimen in HIV-infected patients.

Several studies have examined the use of oral fluconazole in lieu of amphotericin B for initial therapy of cryptococcal meningitis in patients with HIV infection. Larsen and colleagues studied 21 patients and found that amphotericin B plus oral flucytosine resulted in fewer clinical failures and faster cryptococcal clearance of the cerebrospinal fluid than fluconazole alone (14). A large collaborative trial compared results with a relatively low dose of intravenous amphotericin B to those with oral fluconazole and found no significant difference in the overall mortality rate (15). However, in this trial, the early mortality rate, defined as death within the first 2 weeks of therapy, was slightly higher, and time to first negative CSF culture was somewhat longer among patients in the fluconazole group.

The recurrence of cryptococcosis, even after initial therapy has rendered the CSF culture sterile, is extremely common in HIV-infected patients (16); continued antifungal therapy appears to reduce the risk for recurrence (12, 16). Fluconazole in daily doses has been shown to be effective in preventing relapse (16) and is superior to amphotericin B in weekly doses (17).

Current information suggests that therapy for cryptococcal meningitis in HIV-infected patients should begin with amphotericin B, with or without flucytosine. Suppressive therapy with fluconazole should be given subsequently to prevent a relapse. When available, the results of a study sponsored by the Mycoses Study Group and AIDS Clinical Treatment Group of the National Institute of Allergy and Infectious Diseases, National Institutes of Health, should clarify these issues.

### Histoplasmosis, Coccidioidomycosis, and Blastomycosis

Unlike candidiasis and cryptococcosis, infections caused by *Histoplasma capsulatum*, *Coccidioides immitis*, and *Blastomyces dermatitidis* are acquired in specific geographic regions. Infections due to these fungi were not initially associated with HIV infection because the HIV epidemic in the United States began in the large urban areas of the East and West Coasts, outside the areas in which these fungi are endemic. As HIV infection spread to the Midwest, where histoplasmosis and blastomycosis are endemic, and to the Southwest, where coccidioidomycosis occurs, these fungi became recognized as major opportunistic agents.

### Histoplasmosis

*H. capsulatum* var. *capsulatum* causes infection worldwide and is the organism most associated with disease in HIV-infected patients. A few cases of histoplasmosis in HIV-infected patients have been due to *H. capsulatum* var. *duboisii* (18), which is found in tropical Africa. In the Western Hemisphere, infection is concentrated in the eastern United States but is also found in the Caribbean as well as in Central and South America. *H. capsulatum* var. *capsulatum* is typically isolated from soil contaminated with avian or bat excreta, and a number of epidemics among persons without HIV infection have been associated with disruption of contaminated soil.

Most cases of histoplasmosis in patients with HIV infection have occurred within the recognized area for endemic *H. capsulatum* in North America, the Ohio and Mississippi River valleys. However, within that area, great variability has been seen in the incidence of disease, with most cases being reported from a single city, Indianapolis, Indiana (19, 20). Since 1978, Wheat has recorded several outbreaks of histoplasmosis in that city, mostly centered in areas where active construction led to soil disruption. In the most recent outbreak, patients with AIDS accounted for more than 50% of the culture-proven cases of histoplasmosis (21); these data suggest that many of these cases represent new infection due to recent exposure rather than reactivation of latent disease.

Cases of histoplasmosis and HIV-infection have also been reported well outside the recognized histoplasmosis-endemic areas (22, 23). In some instances, these cases represent reactivation of infection acquired during residence in or travel to disease-endemic regions. In other instances, they appear to represent acute infection after disruption of microfoci of *H. capsulatum* that exist outside the recognized disease-endemic areas (19).

Patients with progressive disseminated histoplasmosis, the most common form of disease among HIV-infected persons, usually have fever, malaise, and weight loss over a period of weeks. Diagnosis is often established by isolating the fungus from respiratory secretions, blood, or bone marrow, but detecting *Histoplasma capsulatum* var. *capsulatum* polysaccharide antigen (HPA) in the serum or urine is also helpful (19).

Amphotericin B therapy is usually effective for progressive disseminated histoplasmosis in patients with HIV infection. Itraconazole may be useful for less severe cases (24). However, relapses are extremely common when therapy is stopped (19, 25), and maintenance therapy with intermittent amphotericin B (25) or with itraconazole (26) is required to prevent this. Fluconazole is less effective but can be used by those who cannot tolerate amphotericin B or itraconazole (27). Urine and serum HPA levels decline with successful therapy and can be useful in determining a patient's response to therapy as well as assessing a patient for relapse (26-28).

### Coccidioidomycosis

Within the disease-endemic area (U.S. Southwest), coccidioidomycosis is one of the most frequent opportunistic infections in persons with AIDS (29). In a prospective study in Tucson, Arizona, active coccidioidomycosis developed in 25% of a cohort of HIV-infected patients over a 41-month period (30). The major risk for developing disease was immunosuppression, as manifested by a CD4 lymphocyte count below 250/µl, a diagnosis of AIDS, or anergy indicated on control skin tests. Length of time in the disease-endemic area, a history of prior coccidioidomycosis, and a positive coccidioidal skin test were not associated with the development of active coccidioidomycosis. These data suggest that most coccidioidomycosis cases among HIV-infected persons in a disease-endemic area are recently acquired and not due to reactivation of latent infection. However, as with histoplasmosis, in a small number of HIV-infected patients, previously acquired infection is reactivated, and clinical coccidioidomycosis develops while the patient is residing outside the disease-endemic area (29).

Despite recent outbreaks of coccidioidomycosis in the San Joaquin Valley and in Northridge, California (31, 32), most cases of coccidioidomycosis among HIV-infected patients have been reported from Arizona, particularly the metropolitan areas of Phoenix and Tucson (30, 33). Whether this represents underreporting of disease in California or a true increase in incidence in Arizona is unknown.

Most HIV-infected patients with coccidioidomycosis seek treatment for pulmonary disease. In many, chest radiographs show a diffuse, reticulonodular pattern. Approximately 70% of patients with this pattern die within 1 month despite antifungal therapy (30, 33). In fact, this radiographic pattern may mimic that seen with *Pneumocystis carinii* pneumonia (34). Sites of dissemination frequently seen in patients without HIV infection, such as skin, soft tissue, bone, joint, and meninges, appear less common among patients with HIV infection (30, 33). Serologic tests can be useful in diagnosing coccidioidomycosis in HIV-infected patients, although they are more likely to have negative results than patients without HIV infection (35). On the other hand, a positive coccidioidal complement-fixation serologic test result in an HIV-infected patient, even in the absence of clinical illness, predicts impending active coccidioidomycosis (36).

Comparative trials regarding the therapy of coccidioidomycosis in HIV-infected patients have not been carried out. For severe disease, such as for diffuse, reticulonodular pneumonia, amphotericin B is recommended. However, for less fulminant infection, fluconazole has been effective. As with cryptococcosis and histoplasmosis, life-long maintenance therapy with an oral azole is recommended, although relapses have occurred despite this.

### Blastomycosis

Blastomycosis is the least common of the three endemic mycoses in North America among patients with HIV infection; fewer than 25 cases have been reported. Only recently has an environmental link been established for infection, when the organism was isolated from riverbank soil in association with an outbreak (37).

Pappas and colleagues have published the largest series of cases associated with HIV infection, consisting of 15 patients from 10 medical centers (38). Ten of the 15 cases were reported from sites within the known disease-endemic area, the midwestern and southeastern United States, and the other five patients had resided within the disease-endemic area at some time before the diagnosis. Most patients were clinically immunodeficient at the time of diagnosis and had either chronic pulmonary infection or disease disseminated beyond the lungs, including meningitis. In all but one case, the diagnosis was established by culture of *B. dermatitidis*, whereas results of serologic tests, when performed, were uniformly negative. Both amphotericin B and ketoconazole were used successfully as therapy in the series.

### Aspergillosis

Although disseminated aspergillosis was originally listed as an infection at least moderately predictive of AIDS, it was removed from the list in 1984 because only three cases had been reported among 1,762 patients (39). However, over the last 5 years, the number of cases of invasive aspergillosis among HIV-infected patients has dramatically increased (40-44); more than 75 cases now have been documented. The largest series, containing 33 patients, was reported by Lortholary and colleagues from France (43). All patients had AIDS and a median CD4 lymphocyte count of 27/µl. About half the patients had the traditional risk factors for invasive aspergillosis (neutropenia or corticosteroid use) at the time of diagnosis. Culture of fluid from bronchoalveolar lavage was positive in all cases in which it was done; *A. fumigatus* grew in 29 of 31 patients. In this study, isolating the fungus from bronchoalveolar lavage fluid correlated with histologic evidence of invasive aspergillosis in 14 of 15 patients.

In all series, the death rate has been extremely high despite therapy. Intravenous amphotericin B has been used most often for treatment, but itraconazole has been tried in some instances (40, 43). In a recent study of the use of itraconazole for the treatment of invasive aspergillosis in a variety of patients, therapy was unsuccessful in all 16 patients with AIDS and aspergillosis (45). In seven of these, either toxicity to the drug developed or clinical symptoms worsened while they were receiving itraconazole; nine died, two directly of aspergillosis and seven of other causes.

If the incidence of aspergillosis is increasing among HIV-infected patients, the factors associated with this increase remain unclear. The identified risk factors for aspergillosis have no doubt increased among HIV-infected patients in the last decade with the use of such drugs as zidovudine and ganciclovir, which are associated with neutropenia, and corticosteroids for the treatment of severe *Pneumocystis carinii* pneumonia and other conditions. Others have postulated that prior pneumonia, which may result in diminished macrophage function (43) and contaminated air, inhaled during marijuana smoking (40), may also play a role. Further studies are needed to clarify this.

### Penicilliosis

Piehl and colleagues reported the first case of infection due to *Penicillium marneffei* in an AIDS patient in 1988 when they described a patient in Chicago with persistent fever, anorexia, and a papular skin rash. The patient's travel history was not given. Blood, bone marrow, sputum, and skin biopsy specimen cultures all grew *P. marneffei*. The patient responded to therapy with amphotericin B, but relapsed once the therapy was discontinued (46).

Since that report, the number of reported cases among HIV infected patients has risen rapidly, particularly in association with the HIV epidemic in Thailand. In a series of 80 patients reported by Supparatpinyo and colleagues (47), most patients were men from the Chiang Mai province of northern Thailand. The most common characteristic in these patients was a generalized papular rash; some of the lesions had central ubilication reminiscent of molluscum contagiosum. Diagnosis was usually established by examining Wright-stained samples of bone marrow aspirates or touch smears of skin biopsy specimens.

Treatment with amphotericin B has been successful in most patients. Itraconazole and fluconazole may also be useful. The mortality rate appears most related to a delay in diagnosis. Relapse is common once therapy is stopped (48); therefore, antifungal therapy should be life-long in the HIV-infected patient with penicilliosis.

In northern Thailand, penicilliosis is now the third most common opportunistic infection (after tuberculosis and cryptococcosis) among HIV-infected persons (48). Given that *P. marneffei* appears endemic in Southeast Asia, penicilliosis can be expected to become an even larger problem as the HIV epidemic continues to expand in this part of the world.

### Prevention of Fungal Infections in HIV-Infected Patients

With the rise of fungi as opportunistic pathogens among patients infected with HIV and with the success of preventive therapy for other opportunists, such as *P. carinii*, primary prevention of fungal disease in HIV-infected patients should be pursued. The goal of any such prevention would be to increase both the length and the quality of the patient's life.

Since the availability of the oral azoles, using antifungal agents to prevent fungal diseases in HIV-infected patients has become a promising approach. However, several questions must be considered before antifungal chemoprophylaxis is used (49). Is the prevalence of disease high enough to make the drug useful in most patients? Is the efficacy of the drug in preventing disease sufficient? Does the therapy induce the development of drug resistance? Is the cost of the drug reasonable? Finally, does the drug have an acceptable toxicity profile, and does it interact or interfere with the metabolism of other drugs?

Few answers to these questions are available. In a recently published prospective, randomized study comparing fluconazole to topical clotrimazole (13), fluconazole was significantly better than clotrimazole in preventing oropharyngeal and esophageal candidiasis as well as cryptococcal meningitis. The benefit of fluconazole was greatest among patients whose CD4 lymphocyte count was < 50/µl. However, 10% of the patients had at least one episode of candidiasis while taking fluconazole, which suggests drug resistance. Moreover, the overall and fungal-disease-related death rates were no different between the two groups. Given the present data, further studies will be needed before the issue of primary prevention of fungal disease through chemoprophylaxis is settled (50).

*Dr. Ampel is an associate professor of medicine at the University of Arizona College of Medicine and director of HIV Clinical Services at the Tucson Veterans Affairs Medical Center. His research focuses on the immune response in coccidioidomycosis.*

### References

1. Sangeorzan JA, Bradley SF, He X, Zarins LT, et al. Epidemiology of oral candidiasis in HIV-infected patients: colonization, infection, treatment, and emergence of fluconazole resistance. Am J Med 1994; 97:339-46.
2. Powderly WG, Robinson K, Keath EJ. Molecular epidemiology of recurrent oral candidiasis in human immunodeficiency virus-positive patients: evidence for two patterns of recurrence. J Infect Dis 1993; 168:463-6.
3. Whelan WL, Kirsch DR, Kwon-Chung KJ, Wahl SM, et al. *Candida albicans* in patients with the acquired immunodeficiency syndrome: absence of a novel or hypervirulent strain. J Infect Dis 1990; 162:513-8.
4. Klein RS, Harris CA, Butkus Small C, Moll B, et al. Oral candidiasis in high-risk patients as the initial manifestation of the acquired immunodeficiency syndrome. N Engl J Med 1984; 311:354-8.
5. Imam N, Carpenter CC, Mayer KH, Fisher A, et al. Hierarchical pattern of mucosal *Candida* infections in HIV-seropositive women. Am J Med 1990; 89:142-6.
6. Stevens DA, Greene SI, Lang OS. Thrush can be prevented in patients with acquired immunodeficiency syndrome and the acquired immunodeficiency syndrome-related complex: randomized, double-blind, placebo-controlled study of 100-mg oral fluconazole daily. Arch Intern Med 1991; 151:2458-64.
7. Redding S, Smith J, Farinacci G, Rinaldi M, et al. Resistance of *Candida albicans* to fluconazole during treatment of oropharyngeal candidiasis in a patient with AIDS: documentation by in vitro susceptibility testing and DNA subtype analysis. Clin Infect Dis 1994; 18:240-2

8. Rex JH, Pfaller MA, Rinaldi MG, Polack A, et al. Antifungal susceptibility testing. Clin Microbiol Rev 1993; 6:357-81.
9. Dismukes WE. Cryptococcal meningitis in patients with AIDS. J Infect Dis 1988; 157:624-8.
10. Levitz SM. The ecology of *Cryptococcus neoformans* and the epidemiology of cryptococcosis. Rev Infect Dis 1991; 13:1163-9.
11. Varma A, Swinne D, Staib F, Bennett JE, et al. Diversity of DNA fingerprints in *Cryptococcus neoformans*. J Clin Microbiol 1995; 33:1807-14.
12. Chuck SL, Sande MA. Infections with *Cryptococcus neoformans* in the acquired immunodeficiency syndrome. N Engl J Med 1989; 321:793-9.
13. Powderly WG, Finkelstein D, Feinberg J, Frame P, et al. (NIAID AIDS Clinical Trials Group). A randomized trial comparing fluconazole with clotrimazole troches for the prevention of fungal infections in patients with advanced human immunodeficiency virus infection. N Engl J Med 1995; 332:700-5.
14. Larsen RA, Leal MAE, Chan LS. Fluconazole compared with amphotericin B plus flucytosine for cryptococcal meningitis in AIDS: a randomized trial. Ann Intern Med 1990; 113:183-7.
15. Saag MS, Powderly WG, Cloud GA, Robinson P, et al. (NIAID Mycoses Study Group and the AIDS Clinical Trials Group). Comparison of amphotericin B with fluconazole in the treatment of acute AIDS-associated cryptococcal meningitis. N Engl J Med 1992; 326:83-9.
16. Bozzette SA, Larsen RA, Chiu J, Leal MAE, et al. A placebo-controlled trial of maintenance therapy with fluconazole after treatment of cryptococcal meningitis in the acquired immunodeficiency syndrome. N Engl J Med 1991; 324:580-4.
17. Powderly WG, Saag MS, Cloud GA, Robinson P, et al. A controlled trial of fluconazole or amphotericin B to prevent relapse of cryptococcal meningitis in patients with the acquired immunodeficiency syndrome. N Engl J Med 1992; 326:793-9.
18. Chandenier J, Goma D, Moyen G, Samba-Lefebvre MC, et al. [African histoplasmosis due to *Histoplasma capsulatum* var. *duboisii*: relationship with AIDS in recent Congolese cases]. Sante 1995; 5:227-34.
19. Wheat LJ, Connolly-Stringfield PA, Baker RL, Curfman MF, et al. Disseminated histoplasmosis in the acquired immune deficiency syndrome: clinical findings, diagnosis and treatment, and review of the literature. Medicine [Baltimore] 1990; 69:361-74.
20. Wheat LJ, Slama TG, Zeckel ML. Histoplasmosis in the acquired immune deficiency syndrome. Am J Med 1985; 78:203-10.
21. Wheat LJ. Histoplasmosis in Indianapolis. Clin Infect Dis 1992; 14 (Suppl 1):S91-9.
22. Huang CT, McGarry T, Cooper S, Saunders R, et al. Disseminated histoplasmosis in the acquired immunodeficiency syndrome: report of five cases from a nonendemic area. Arch Intern Med 1987; 147:1181-4.
23. Salzman SH, Smith RL, Aranda CP. Histoplasmosis in patients at risk for the acquired immunodeficiency syndrome in a nonendemic setting. Chest 1988; 93:916-21.
24. Wheat J, Hafner R, Korzun AH, Limjoco MT, et al. (AIDS Clinical Trials Group). Itraconazole treatment of disseminated histoplasmosis in patients with the acquired immunodeficiency syndrome. Am J Med 1995; 98:336-42.
25. McKinsey DS, Gupta MR, Riddler SA, Driks MR, et al. Long-term amphotericin B therapy for disseminated histoplasmosis in patients with the acquired immunodeficiency syndrome (AIDS). Ann Intern Med 1989; 111:655-9.
26. Wheat J, Hafner R, Wulfsohn M, Spencer P, et al. (NIAID Clinical Trials and Mycoses Study Group Collaborators). Prevention of relapse of histoplasmosis with itraconazole in patients with the acquired immunodeficiency syndrome. Ann Intern Med 1993; 118:610-6.
27. Norris S, Wheat J, McKinsey D, Lancaster D, et al. Prevention of relapse of histoplasmosis with fluconazole in patients with the acquired immunodeficiency syndrome. Am J Med 1994; 96:504-8.
28. Wheat LJ, Connolly-Stringfield P, Blair R, Connolly K, et al. Effect of successful treatment with amphotericin B on *Histoplasma capsulatum* variety *capsulatum* polysaccharide antigen levels in patients with AIDS and histoplasmosis. Am J Med 1992; 92:153-60.
29. Jones JL, Fleming PL, Ciesielski CA, Hu DJ, et al. Coccidioidomycosis among persons with AIDS in the United States. J Infect Dis 1995; 171:961-6.
30. Ampel NM, Dols CL, Galgiani JN. Coccidioidomycosis during human immunodeficiency virus infection: results of a prospective study in a coccidioidal endemic area. Am J Med 1993; 94:235-40.
31. Einstein HE, Johnson RH. Coccidioidomycosis: new aspects of epidemiology and therapy. Clin Infect Dis 1993; 16:349-56.
32. CDC. Coccidioidomycosis following the Northridge Earthquake—California, 1994. MMWR 1994; 43:194-5.
33. Fish DG, Ampel NM, Galgiani JN, Dols CL, et al. Coccidiodiomycosis during human immunodeficiency virus infection: a review of 77 patients. Medicine [Baltimore] 1990; 69:384-91.
34. Mahaffey KW, Hippenmeyer CL, Mandel R, Ampel NM. Unrecognized coccidioidomycosis complicating *Pneumocystis carinii* pneumonia in patients infected with the human immunodeficiency virus and treated with corticosteroids: a report of two cases. Arch Intern Med 1993; 153:1496-8.
35. Antoniskis D, Larsen RA, Akil B, Rarick MU, et al. Seronegative disseminated coccidioidomycosis in patients with HIV infection. AIDS 1990; 4:691-3.
36. Arguinchona HL, Ampel NM, Dols CL, Galgiani JN, et al. Persistent coccidioidal seropositivity without clinical evidence of active coccidioidomycosis in patients infected with human immunodeficiency virus. Clin Infec Dis 1995; 20:1281-5.
37. Klein BS, Vergeront JM, Weeks RJ, Kumar UN, et al. Isolation of *Blastomyces dermatitidis* in soil associated with a large outbreak of blastomycosis in Wisconsin. N Engl J Med 1986; 314:529-34.
38. Pappas PG, Pottage JC, Powderly WG, Fraser VJ, et al. Blastomycosis in patients with the acquired immunodeficiency syndrome. Ann Intern Med 1992; 116:847-53.
39. Jaffe HW, Selik RM. Acquired immunodeficiency syndrome: is disseminated aspergillosis predictive of underlying cellular deficiency? [letter]. J Infect Dis 1984; 149:829.

40. Denning DW, Follansbee SE, Scolaro M, Norris S, et al. Pulmonary aspergillosis in the acquired immunodeficiency syndrome. N Engl J Med 1991; 324:654-62.
41. Singh N, Yu VL, Rihs JD. Invasive aspergillosis in AIDS. South Med J 1991; 84:822-6.
42. Klapholz A, Salomon N, Perlman DC, Talavera W. Aspergillosis in the acquired immunodeficiency syndrome. Chest 1991; 100:1614-8.
43. Lortholary O, Meyohas MC, Dupont B, Cadranel J, et al. (French Cooperative Study Group on Aspergillosis in AIDS). Invasive aspergillosis in patients with acquired immunodeficiency syndrome: report of 33 cases. Am J Med 1993; 95:177-87.
44. Miller WT, Jr., Sais GJ, Frank I, Gefter WB, et al. Pulmonary aspergillosis in patients with AIDS: clinical and radiographic correlations. Chest 1994; 105:37-44.
45. Denning DW, Lee JY, Hostetler JS, Pappas P, et al. NIAID Mycoses Study Group multicenter trial of oral itraconazole therapy for invasive aspergillosis. Am J Med 1994; 97:135-44.
46. Piehl MR, Kaplan RL, Haber MH. Disseminated penicilliosis in a patient with acquired immunodeficiency syndrome. Arch Pathol Lab Med 1988; 112:1262-4.
47. Supparatpinyo K, Sirisanthana T. Disseminated *Penicillium marneffei* infection diagnosed on examination of a peripheral blood smear of a patient with human immunodeficiency virus infection. Clin Infect Dis 1994; 18:246-7.
48. Supparatpinyo K, Khamwan C, Baosoung V, Nelson KE, et al. Disseminated *Penicillium marneffei* infection in southeast Asia. Lancet 1994; 344:110-3.
49. Perfect JR. Antifungal prophylaxis: to prevent or not? Am J Med 1993; 94:233-4.
50. Powderly WG. Prophylaxis for HIV-related infections: a work in progress. Ann Intern Med 1996; 124:342-4.
